# Neuronally-derived tau is increased in experienced breachers and is associated with neurobehavioral symptoms

**DOI:** 10.1038/s41598-021-97913-0

**Published:** 2021-09-30

**Authors:** Katie A. Edwards, Kisha Greer, Jacqueline Leete, Chen Lai, Christina Devoto, Bao-Xi Qu, Angela M. Yarnell, Elena Polejaeva, Kristine C. Dell, Matthew L. LoPresti, Peter Walker, Eric M. Wassermann, Walter Carr, James R. Stone, Stephen T. Ahlers, Rany Vorn, Carina Martin, Jessica M. Gill

**Affiliations:** 1 National Institutes of Health, National Institute of Nursing Research, Bethesda, MD 20814 USA; 2 Henry M. Jackson Foundation for the Advancement of Military Medicine, Bethesda, MD 20817 USA; 3 Military Emergency Medicine Department, Uniformed Services University, Bethesda, MD 20814 USA; 4 Department of Clinical and Health Psychology, University of Florida, Gainsville, FL 32603 USA; 5 Department of Psychology, Pennsylvania State University, University Park, PA 16801 USA; 6 Center for Military Psychiatry and Neuroscience, Walter Reed Army Institute of Research, Silver Spring, MD 20910 USA; 7 Joint Artificial Intelligence Center, Arlington, VA 2220 USA; 8 National Institutes of Health, National Institute of Neurological Disorders and Stroke, Bethesda, MD 20814 USA; 9 Oak Ridge Institute for Science and Education, Oak Ridge, TN 37830 USA; 10 Department of Radiology and Medical Imaging, University of Virginia, Charlottesville, VA 22903 USA; 11 Operational and Undersea Medicine Directorate, Naval Medical Research Center, Silver Spring, MD 20910 USA; 12 Center for Neuroscience and Regenerative Medicine, Uniformed Services University of the Health Sciences, Bethesda, MD 20814 USA

**Keywords:** Biomarkers, Neurological disorders

## Abstract

Military and law enforcement breachers are exposed to many low-level blasts during their training and occupational experiences in which they detonate explosives to force entry into secured structures. There is a concern that exposure to these repetitive blast events in career breachers could result in cumulative neurological effects. This study aimed to determine concentrations of neurofilament light (NF-L), tau, and amyloid-beta 42 (Aβ42) in serum and in neuronal-derived extracellular vesicles (EVs) in an experienced breacher population, and to examine biomarker associations with neurobehavioral symptoms. Thirty-four participants enrolled in the study: 20 experienced breachers and 14 matched military or civilian law enforcement controls. EV tau concentrations were significantly elevated in experienced breachers (0.3301 ± 0.5225) compared to controls (−0.4279 ± 0.7557; F = 10.43, p = 0.003). No statistically significant changes were observed in EV levels of NF-L or Aβ42 or in serum levels of NF-L, tau, or Aβ42 (p’s > 0.05). Elevated EV tau concentrations correlated with increased Neurobehavioral Symptom Inventory (NSI) score in experienced breachers (r = 0.596, p = 0.015) and predicted higher NSI score (*F*(1,14) = 7.702, *p* = 0.015, *R*^*2*^ = 0.355). These findings show that neuronal-derived EV concentrations of tau are significantly elevated and associated with neurobehavioral symptoms in this sample of experienced breachers who have a history of many low-level blast exposures.

## Introduction

Blast injury is a critical concern for military forces due to the use of improvised explosive devices in the Iraq and Afghanistan conflicts^1^. In contrast to large magnitude explosives in combat, experienced breachers in military and law enforcement professions encounter hundreds to thousands of training and occupational exposures to low-level blast overpressure over the course of their careers. Breachers construct and detonate explosives, often while at a close distance, to create a breach in a locked door or wall to force entry into a secured structure. While these subconcussive blast exposures do not result in medically diagnosed injuries, there may be neurological sequelae due to the chronic, repetitive nature of these exposures. Research suggests that repeated low-level blast exposure is associated with neurological effects, including cognitive, somatic, and affective symptoms as well as neurocognitive and neurosensory decline and potentially increased vulnerability to diagnosable neurological injury^2–6^. It is important to further examine the risks of developing neurological symptoms following blast exposure in this unique population as well as elucidate the underlying biological mechanisms that relate to these associated symptoms.

Blood-based biomarkers are promising as potential objective indicators to identify blast exposed individuals who may be at risk for neurological symptoms and/or other deficits. In military and civilian populations, traumatic brain injuries (TBIs) have been linked to increased levels of neuronal proteins neurofilament-light (NF-L), tau, and amyloid beta-42 (Aβ42), and these increases have further been linked to symptom development^7–12^. Moreover, repeated exposure to low-level blasts has been associated with a reduction in neurocognitive performance that correlated with elevations in serum levels of NF-L, tau, and Aβ42^13^.

One important issue that has been raised in TBI blood-based biomarker research is the identification of neuronal effects. Extracellular vesicles (EVs) are nanoparticles with a lipid bilayer membrane that carry proteins, genetic material, and lipids from inside of the cell, participate in cell-to-cell communication, and are readily measured in peripheral circulation^14^. EVs protect proteins from degradation and may be a stable source of brain-related proteins^15–18^. Our lab identified elevated levels of GFAP in neuronal-derived EVs of TBI patients^19^, while lower serum GFAP levels were observed following blast exposure in a military training population^20^. These findings suggest that neuronal-derived EVs have a heightened specificity for central nervous system (CNS) processes, potentially increasing clinical relevance for neurological outcomes. However, the clinical relevance of serum versus neuronal-derived EV concentrations of NF-L, tau, and Aβ42 levels in experienced breacher populations remains elusive.

To address this critical issue, we analyzed concentrations of NF-L, tau, and Aβ42 in serum and in neuronal-derived EVs in an experienced breacher population with a high number of low-level blast exposures and examined if these biomarkers associate with neurobehavioral symptoms.

## Results

### Demographics and clinical characteristics

Thirty-four males were recruited into the study (20 breachers and 14 matched military or civilian law enforcement controls). Overall blast exposures ranged from 456 to 35,800 exposures in the breacher group and from 0 to 39 exposures in the control group. The majority of participants were white (85%) military personnel (65–71%). Participants’ ages ranged from 26 to 54 years, with a mean age of 39 years. There were no statistically significant differences in demographics, prior service, Beck Depression Inventory (BDI) or Neurobehavioral Symptom Inventory (NSI) between experienced breachers and controls (Table 1). There were higher reported PCL-M scores in experienced breachers when compared with controls, indicating increased PTSD-related symptoms, although these levels do not meet clinical criteria for PTSD diagnosis.

**Table 1:** Demographics and clinical characteristics. Data is described using mean and SD.

	Experienced breacher (N = 20)	Unexposed control (N = 14)	Significance
**Race, no. (%)**			χ^2^ = 2.893 p = 0.576
White	17 (85)	12 (85.71)	
Black	0 (0)	1 (7.14)	
Asian/Pacific Islander	1 (5)	1 (7.14)	
American Indian/Alaskan	1 (5)	0 (0)	
Other	1 (5)	0 (0)	
Ethnicity (non-Hispanic), no. (%)	19 (95)	13 (92.86)	χ^2^ = 0.068 p = 0.794
Sex (male), no. (%)	20 (100)	14 (100)	N/A
**Type of service, no. (%)**			χ^2^ = 2.839 p = 0.242
Military	14 (65)	10 (71.43)	
Civilian law enforcement	3 (20)	4 (28.57)	
Both	3 (15)	0 (0)	
**Age (years)**			
Mean age (SD)	39.65 (8.337)	38.86 (7.814)	t = 0.280 p = 0.781
Minimum–maximum	26–54	27–53	
Mean years of education (SD)	14.25 (1.743)	14.43 (2.593)	t = -0.241 p = 0.811
Mean years of service (SD)	16.80 (6.693)	13.92 (6.986)	t = 1.209 p = 0.235
**Number of prior blast exposures, no. (%)**			N/A
* < 40*	0 (0)	14 (100)	
*400 + *	20 (100)	0 (0)	
*Minimum–maximum*	456–34,800	0–39	
**Most recent blast exposure, no. (%)**			χ^2^ = 29.046 p < 0.01
Never	0 (0)	11 (78.57)	
Past week	4 (20)	0 (0)	
Past month	8 (40)	0 (0)	
Past 6 months	3 (15)	0 (0)	
Past year	3 (15)	0 (0)	
More than 1 year	2 (5)	3 (21.43)	
Mean number of self-reported head injuries (SD)	0.80 (0.616)	0.36 (0.497)	t = 2.228 p = 0.033
BDI (SD)	4.25 (4.518)	3.00 (5.038)	t = 0.757 p = 0.899
PCL-M (SD)	25.55 (6.924)	20.64 (4.483)	t = 2.327 p = 0.027
NSI (SD)	16.90 (5.955)	16.86 (5.289)	t = 0.022 p = 0.824

*BDI* Beck Depression Index, *PCL-M* Post-Traumatic Stress Disorder Checklist-Military, *NSI* Neurobehavioral Symptom Inventory.

### Protein, neuronal-derived EV, and psychometric testing

**Figure 1:**
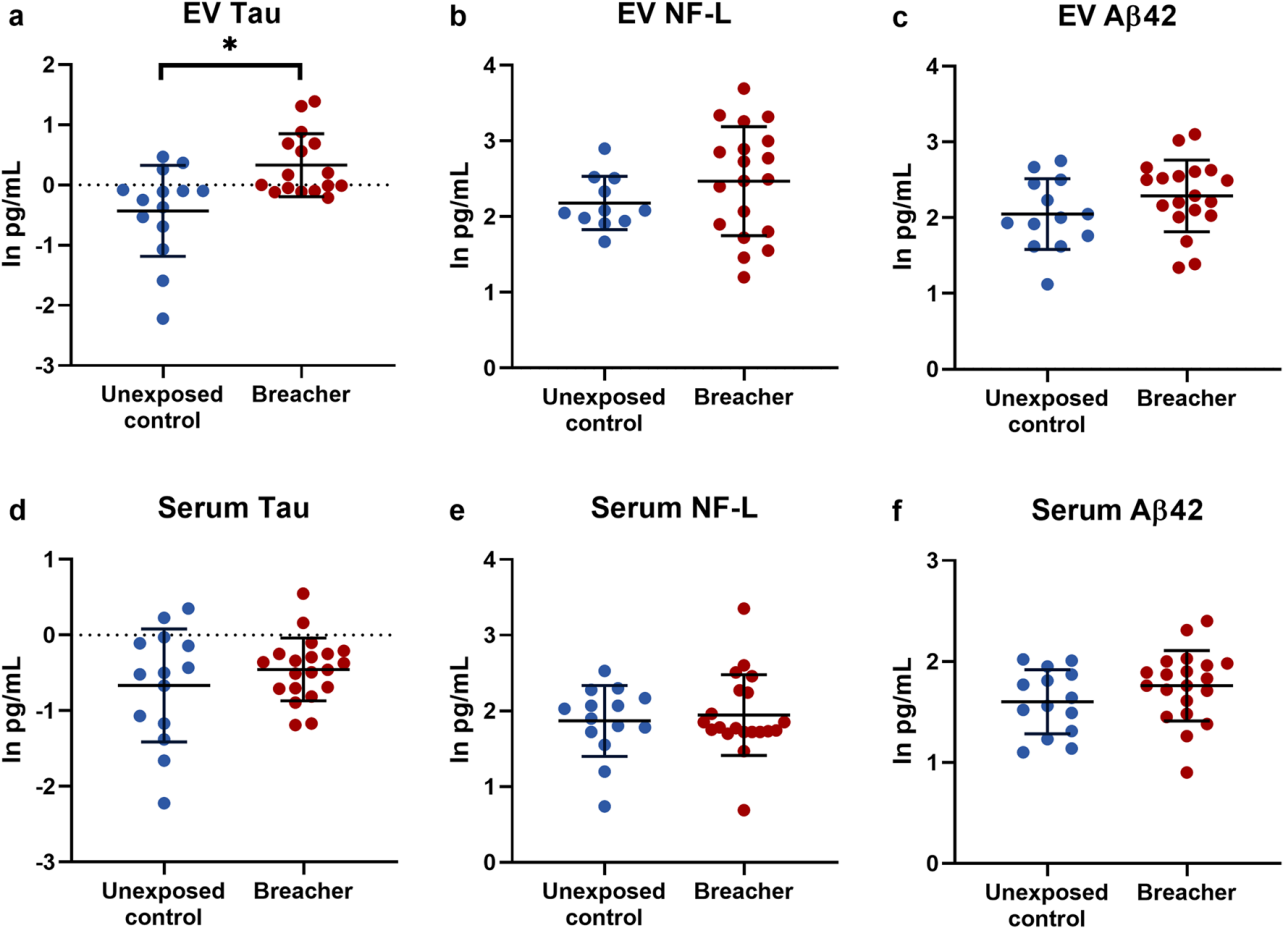
Neuronal-derived EV tau distinguishes experienced breachers from controls. **(a)** Exosomal tau was elevated in the experienced breacher group compared to the control group. There were no statistically significant changes in EV levels of **(b)** NF-L or **(c)** Aβ42. There were no statistically significant changes in serum levels of **(d)** tau, **(e)** NF-L, or **(f)** Aβ42. *indicates p < 0.05, error bars represent the mean and SD. *NF-L* neurofilament light, *Aβ* amyloid-beta.

Neuronal-derived EV tau concentrations (natural log-transformed mean ± SD) were significantly elevated in experienced breachers (0.3301 ± 0.5225) compared to controls (−0.4279 ± 0.7557; F = 10.43, p = 0.003) (Fig. 1a). No statistically significant changes were noted in EV NF-L (p = 0.224) or Aβ42 (p = 0.168) (Fig. 1b,c). There were no statistically significant differences between groups in serum levels of NF-L (p = 0.672), tau (p = 0.302), or Aβ42 (p = 0.190) (Fig. 1d–f).

Elevated EV tau levels significantly correlated with increased NSI score in experienced breachers (r = 0.596, p = 0.015) (Table 2). In linear regression, EV tau significantly predicted higher NSI score (*F*(1,14) = 7.702, *p* = 0.015, *R*^*2*^ = 0.355).

**Table 2:** Biomarker spearman correlations with psychometric tests.

		Serum NF-L	Serum Tau	Serum Aβ42	EV NF-L	EV Tau	EV Aβ42
BDI	Pearson coefficient	0.367	0.055	0.281	0.363	−0.131	−0.106
	p-value	0.111	0.819	0.229	0.126	0.627	0.665
	N	20	20	20	19	16	19
PCL-M	Pearson coefficient	0.302	0.231	0.277	0.266	−0.496	0.050
	p-value	0.196	0.328	0.237	0.272	0.051	0.840
	N	20	20	20	19	16	19
NSI	Pearson coefficient	−0.066	−0.009	−0.173	0.140	0.596*	−0.310
	p-value	0.783	0.968	0.466	0.568	0.015	0.196
	N	20	20	20	19	16	19

*p < 0.01.

*BDI* Beck Depression Index, *PCL-M* Post-Traumatic Stress Disorder Checklist-Military, *NSI* Neurobehavioral Symptom Inventory.

## Discussion

In this study we report a significant elevation in neuronal-derived EV concentrations of tau. Further, we found that this central measure of tau related to neurobehavioral symptom burden. These findings implicate neuronally-derived tau in repetitive, low-level blast exposures and suggest that this elevation relates to persistent neurological symptoms.

The observation of elevated tau in neuronal-derived EVs aligns with previous reports of EV sources of tau in mild TBI, including repetitive injuries^21,22^. Conversely, our finding that experienced breachers showed no statistically significant changes in serum levels of tau, NF-L, and Aβ42 differs from prior reports of elevated levels in military and civilian TBI populations^7–12^. This discrepancy may be explained by the studied population. Evidence suggests that blast exposure has a distinct neuropathology from the blunt force impacts observed in these aforementioned studies^23^. Prior work in a blast-exposed population also reports elevated serum levels of tau, NF-L, and Aβ42^13^; however, that population was exposed acutely and earlier in their careers, during initial breacher training activities, and did not experience the cumulative exposures in this current population of experienced breachers. Many studies are showing acute effects^2,3,20^, whereas ours assessed the cumulative effects over a career. The acute vs. cumulative (chronic) dimension is important, particularly if it sets the conditions for pathological conditions that take years to develop. These differing results imply not only the importance of stratifying heterogenous blunt impact and blast exposure populations (i.e. experienced breachers), but also the importance of differentiating central and peripheral neuropathological mechanisms.

Tau and Aβ42 are neuronal structure proteins that can be detected in circulation after TBI with elevations linked to TBI severity and clinical outcomes^8,12^. Accumulation of these proteins has well-recognized links to neurodegenerative processes including Alzheimer’s disease and chronic traumatic encephalopathy^24–26^. Following blast exposure, evidence from preclinical models suggests marked disturbance to cerebrovascular functioning^27^, although altered BBB function following blast exposures in human populations remains unclear. Because EVs transport cellular material (including neurotoxic proteins) across the BBB and may contribute to neurodegenerative processes^28^, understanding the impact of neuronal-derived EVs in repetitive blast exposure is needed. Additionally, while tau is predominantly expressed in neurons, tau is not a brain-specific protein and is expressed in other tissues such as muscle fibers^29^. Thus, the ability to distinguish CNS from peripheral sources of tau is essential in the context of understanding the neurological response to repetitive blast exposures and subsequent chronic neurological outcomes; measuring tau in neuronally-derived EVs may be one avenue in which this can be accomplished.

Elevated neuronal-derived EV tau correlates with increased NSI score in the breacher population, implicating neuronal-specific tau with repetitive low-level blast exposures and worse clinical outcomes. This finding suggests clinical relevance considering prior studies in military populations have also reported poor neurocognitive outcomes. Acute blast overpressure has been previously linked to decreased neurocognitive performance^2^, and chronic occupational exposure to low level blasts has been linked to cognitive, affective, and physical symptoms similar to concussion^3^. Brain imaging changes together with cognitive and neurobehavioral deficits have been observed in military veterans with mTBI resulting from repetitive blast exposures^30^. Further, an over twofold increased risk in developing dementia was associated with mTBI in a large retrospective cohort study of 350,000 military personnel and veterans, highlighting an imminent concern for the aging veteran population^31,32^. The link between neurobehavioral symptoms and EV tau levels in breachers implies a combination approach of blood-based biomarkers and psychometric testing may be valuable to identify those who may be at risk for developing adverse long-term outcomes.

A major strength of this study is the comparison of both serum and neuronal-derived EV levels of proteins related to neurodegeneration in a unique population with occupational exposure to repetitive low-level blasts. Importantly, we determined neuronal sources of tau were linked with neurobehavioral outcomes. The recruitment of experienced breacher and control groups with similar occupational environments controls for career-related factors (e.g. physical exertion) between groups and suggests protein changes may be more specific to repetitive blast exposures rather than other occupational factors. This study was constrained by a small sample size. The lack of additional time points precluded a longitudinal comparison of outcomes in relation to the initially reported protein levels. The biodistribution of L1-CAM should be taken into consideration in the interpretation of these findings. Although enriched in neurons^33^, L1-CAM is expressed in both central and peripheral nerve cells^34^, and, as described more recently, in other cell types, including blood and immune cells^35^. Examination of the mechanism causing the observed increase in neuronal-derived EV tau was also outside the scope of this work.

Despite these limitations, our results suggest that examining links between neural proteins and symptoms may have clinical relevance in experienced breacher populations. Future studies should continue to investigate serum and neuronal EV levels of neuronal-related proteins to identify and verify biomarkers that may be indicative of neurological outcomes in experienced breachers.

## Methods

### Clinical methodology

The Institutional Review Boards at the Naval Medical Research Center (NMRC) and the National Institutes of Health (NIH) reviewed and approved all study procedures, and all methods were performed in accordance with guidelines and regulations. All participants provided written informed consent prior to study enrollment. Procedures were implemented at the NIH Clinical Center over a five-day evaluation period. The study enrolled 20 active or prior active duty military or civilian law enforcement breachers with: (1) four or more years of active breaching experience or (2) exposed to greater than 400 breaching blasts during a career but no acute exposures prior to participation in this study. Fourteen controls were enrolled with 4 or more years of military or civilian law enforcement experience, with active involvement in military or civilian law enforcement training or operations, and exposure to 40 or less blasts over their career. Exclusion criteria included: a history of moderate to severe TBI with loss of consciousness greater than 5 min, central nervous system (CNS) disorder, respiratory conditions, cardiac conditions, and any other health conditions influencing cerebral metabolism.

### Demographics, clinical history, and psychometric testing

Demographic and clinical information was gathered during interviews with participants. Psychometric tests were utilized to evaluate cognitive domains and symptomology. The Beck Depression Inventory (BDI) was used to measure depression symptom severity^36^. The BDI is a 21-item self-report rating scale with high internal consistency for both psychiatric and non-psychiatric populations (α = 0.86 and 0.81, respectively)^37^. The Post-Traumatic Stress Disorder Checklist-Military (PCL-M) was administered to screen for combat-related PTSD symptoms. The PCL-M is a 17-item self-report scale. It has been shown to have high test–retest reliability (r = 0.96) and internal consistency (α = 0.96) in Vietnam veterans^38^. The Neurobehavioral Symptom Inventory (NSI) was used to assess postconcussive symptoms. The NSI is a 22-item self-report scale and has shown both excellent internal consistency (α = 0.95) as well as the ability to differentiate veterans with TBIs from those without^39^.

### Blood biomarkers

Non-fasting blood samples were collected between 9:00am and 12:00 pm prior to interviews and the psychometric testing. Peripheral blood samples were drawn into ethylenediaminetetraacetic acid (EDTA) tubes and processed for serum within one hour using standard protocols^11^. Blood samples were stored at −80 °C until batch assay processing.

### Neuronal-derived EV isolation

Total EVs were isolated from frozen serum aliquots and enriched for neuronal-derived EVs as previously described^22^. Briefly, EVs were isolated from 0.5 mL of frozen human serum following ExoQuick manufacturer instructions (System Biosciences, Cat # EXOQ5TM-1, Palo Alto, CA, USA) and MISEV2018 reporting guidelines^40^ as previously described^41^. For EV characterization data, see supplementary material and supplemental fig. S1. Neuronal-derived EVs were enriched by using 4 μg of biotinylated antibodies against neuronal surface markers CD171 (L1CAM) (clone 5G3; eBioscience, San Diego, CA, USA) in 50 μL of 3% Bovine Serum Albumin (BSA) (Thermo-Fisher Scientific Inc., Rockford, IL, USA), and followed by adding 15 μL of Streptavidin-agarose Ultralink resin (Thermo-Fisher Scientific Inc., Rockford, IL, USA) in 25 μL of 3% BSA per tube. The resin pellet was resuspended in 200 μL of 0.1 M glycine–HCl and centrifuged at 4 °C (for 10 min at 4500 × *g*). Supernatant fluid was then harvested to new collecting tubes containing 25 μL of 10% BSA and 15 μL of 1 M Tris–HCl and mixed. Neuronal-derived EVs were lysed with equal volume of mammalian protein extraction reagent (M-PER) (Thermo-Fisher Scientific Inc., Rockford, IL, USA), and then the lysis was ready for downstream analysis.

### Protein quantification

NFL, tau, and Aβ42 proteomic analyses were measured separately from serum samples and neuronal-derived EVs in duplicate using high-definition (HD-1), single-molecule array technology (Simoa, Quanterix, Lexington, MA). The coefficient of variation for all concentration values were < 20%. The lower limit of detection for each assay are: NFL, 0.038 pg/mL; tau, 0.019 pg/mL; and Aβ42, 0.045 pg/mL.

### Statistical analysis

Statistical analysis was conducted with SPSS Build 1.0.0.1298 (Armonk, NY: IBM Corp.). Figures were created using GraphPad Prism version 8.2.0 (La Jolla, CA: GraphPad Software). Demographic and clinical characteristics were compared between the experienced breacher and control groups using chi-square and independent samples t-test. To compare biomarker levels between the experienced breacher and control groups, values were natural log-transformed to adjust for normality. Pearson’s correlations were used to evaluate biomarker values and psychometric tests within the breacher group. Protein concentrations were compared between groups using one-way ANOVA. Linear regression was subsequently run to determine ability of significant biomarkers to predict psychometric testing outcomes within the experienced breacher group. Statistical tests were two-tailed and p < 0.05 was considered a significant difference.

## Supplementary Information

Supplementary Information.

## Data Availability

The anonymized data that support the findings of this study are available upon reasonable request from any qualified investigator to the corresponding author.
