# Supplementary material for: Neuronally-derived tau is increased in experienced breachers and is associated with neurobehavioral symptoms
Source: Sci Rep. 2021 Sep 30;11:19527. doi: 10.1038/s41598-021-97913-0 (PMC8484560; doi:10.1038/s41598-021-97913-0)
Supplement: Supplementary file 1 — Supplementary Information. [file 41598_2021_97913_MOESM1_ESM.docx]

**EV Characterization**

**Methods and Materials**

Human serum samples were subjected to extracellular vesicle (EV) isolation by using ExoQuick kit (System Biosciences, LLC, CA, USA). EV Samples were then subjected to bead-based multiplex EV analysis by using human MACSPlex Exosome Kit (Miltenyi Biotec, Germany) [1]. EV-containing samples were processed as manufacturer indicated and briefly as follows: EV samples equivalent to 20 ug of protein amount or an input dose of ~1 x 10^6^ EVs were diluted with MACSPlex buffer (MPB) to a final volume of 120 µL and loaded onto pre-wet and drained MACSPlex 96-well 0.22 µm filter plate before 15 µL of MACSPlex Exosome Capture Beads (containing 39 different antibody-coated bead subsets) were added to each well. Filter plates were then incubated on an orbital shaker overnight at 450 rpm at room temperature (RT) protected from light. Beads were washed with 200 µL of MPB per well and the filter plate was centrifuged at 300 x g, 3 min. 135 µL of MPB and 5 µL of each APC-conjugated anti-CD9, anti-CD63, and anti-CD81 detection antibody were added per well for counterstaining and the plate was incubated on a shaker at 450 rpm protected from light for 1 h at RT. The plate was then washed by adding 200 µL MPB to each well followed by centrifugation. The plate was again washed with 200 µL of MPB, followed by incubation on a shaker at 450 rpm protected from light for 15 min at RT and centrifugation. Subsequently, 150 µL of MPB was added to each well, and flow cytometric analysis was performed with a MACSQuant Analyzer 10 flow cytometer equipped with 405, 488, and 638 nm lasers (Miltenyi Biotec) by using the built-in 96-well plate reader. All samples were mixed immediately before 100 µL were loaded to and acquired by the instrument, resulting in approximately 20,000 single bead events being recorded per sample. FlowLogic and MACSQuant softwares (Miltenyi Biotec) were used to analyze flow cytometric data. Representative images were shown for all 39 capture bead subsets, or the bead subsets with CD9, CD63, or CD81 positive staining.

**Results**

EVs from the control and breacher serum samples were evaluated by using MACSplex assay, a multiplex bead-based flow cytometry assay platform, which comprises 39 hard-dyed capture bead populations coated with different monoclonal antibodies against 37 potential EV surface antigens or two internal isotype negative controls respectively, followed by counterstaining with a mixture of all three APC-labeled detection antibodies against the tetraspanins CD9, CD63, and CD81, which are three of the most common EV surface markers. After data acquisition, single bead event gating was followed according to the manufacturer’s instruction and applied to all the samples in the same experiments (Figure S1A, D). All 37 capture bead populations could be identified by PE and FITC channel detection in each EV sample, and the results were analyzed according to the assay documentation provided by the manufacturer (Figure S1B, E). Positive APC signal of CD9, CD63, and CD81 staining in EV samples was detected via PE and APC channel detection (Figure S1F), by subtracting corresponding APC background signal obtained from no EV controls (capture beads + detection antibodies) (Figure S1C).

Bead populations of CD9, CD29, CD63, CD69, CD81, and other EV markers after backgating positive APC fluorescence signals were displayed in Figures S1G, which indicated that EV carrying these markers could be the dominant EV populations in the group of samples. Other EV markers detected at low-positive APC fluorescence intensity levels were not shown in backgating events.

**Reference**

1. Wiklander, O.P.B., et al., *Systematic Methodological Evaluation of a Multiplex Bead-Based Flow Cytometry Assay for Detection of Extracellular Vesicle Surface Signatures.* 2018. **9**(1326).


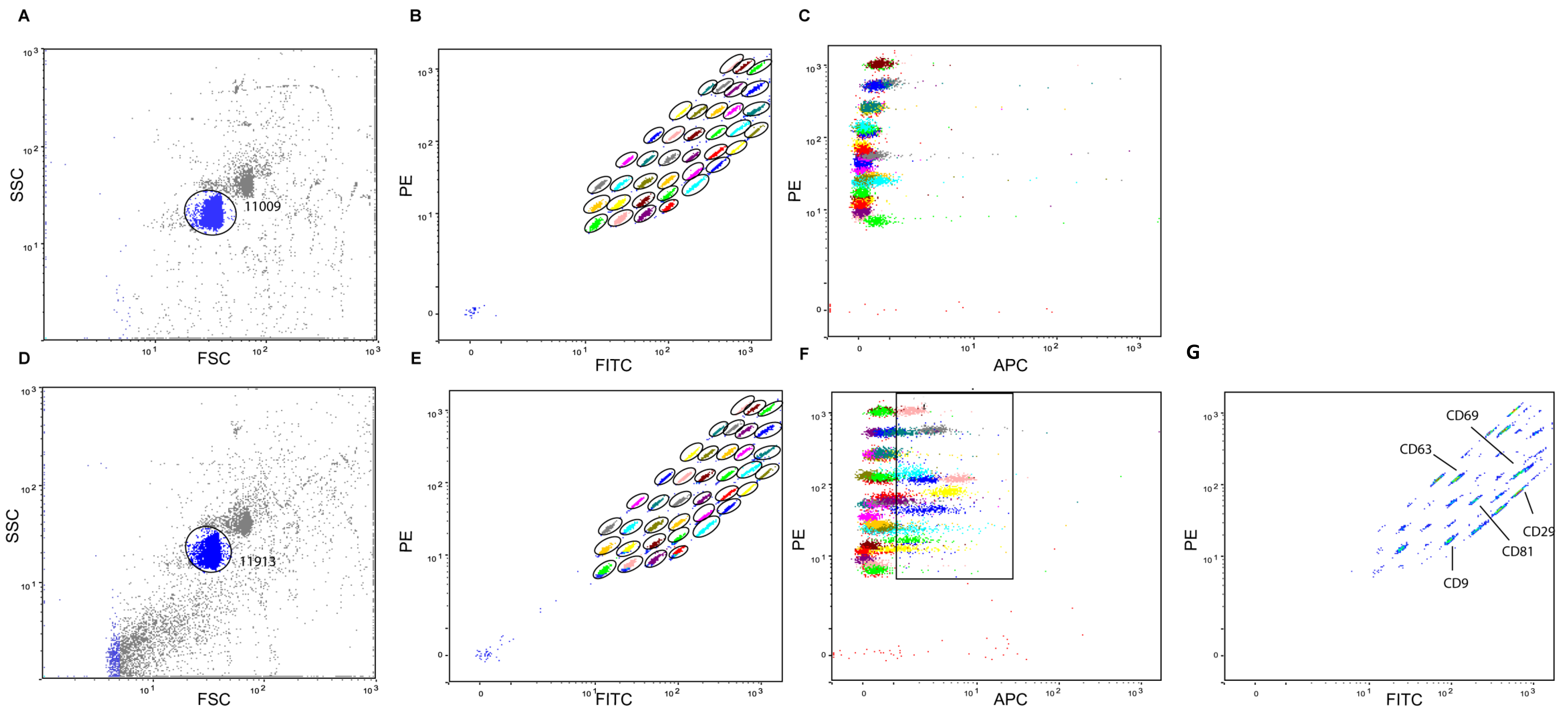


**Supplemental Figure S1.** Representative EV characterization results by using MACSplex exosome kit. Side scatter and forward scatter image for the respective no EV control (A) and EV sample (D). B and E showed all 39 bead populations identified by their fluorescence in the PE Vs. FITC channel of the respective no EV control (B) and EV sample (E). Dot plots showed respective APC-stained bead populations in the respective no EV control (C) and EV sample (F). Back-gating from the most intensive APC positive bead events showed the brightest staining (CD9, CD29, CD63, CD69, and CD81) in the representative EV sample (G).
